# Understanding the role of wild ruminants in anthelmintic resistance in livestock

**DOI:** 10.1098/rsbl.2022.0057

**Published:** 2022-05-04

**Authors:** Tony L. Brown, Paul M. Airs, Siobhán Porter, Paul Caplat, Eric R. Morgan

**Affiliations:** ^1^ School of Biological Sciences, Queen's University Belfast, Belfast, UK; ^2^ Institute for Global Food Security, Queen's University Belfast, Belfast, UK; ^3^ Veterinary Sciences Division, Agri-food and Biosciences Institute, Belfast, UK

**Keywords:** nematodes, *Haemonchus contortus*, trematodes, deer, *refugia*, transmission

## Abstract

Wild ruminants are susceptible to infection from generalist helminth species, which can also infect domestic ruminants. A better understanding is required of the conditions under which wild ruminants can act as a source of helminths (including anthelmintic-resistant genotypes) for domestic ruminants, and vice versa, with the added possibility that wildlife could act as *refugia* for drug-susceptible genotypes and hence buffer the spread and development of resistance. Helminth infections cause significant productivity losses in domestic ruminants and a growing resistance to all classes of anthelmintic drug escalates concerns around helminth infection in the livestock industry. Previous research demonstrates that drug-resistant strains of the pathogenic nematode *Haemonchus contortus* can be transmitted between wild and domestic ruminants, and that gastro-intestinal nematode infections are more intense in wild ruminants within areas of high livestock density. In this article, the factors likely to influence the role of wild ruminants in helminth infections and anthelmintic resistance in livestock are considered, including host population movement across heterogeneous landscapes, and the effects of climate and environment on parasite dynamics. Methods of predicting and validating suspected drivers of helminth transmission in this context are considered based on advances in predictive modelling and molecular tools.

## 1. Introduction

**Figure 1.**
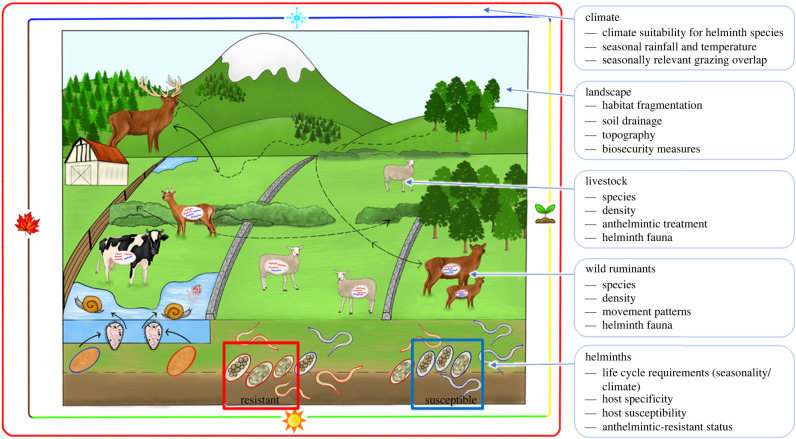
Factors that impact the transmission of generalist helminths, including anthelmintic-resistant strains, between wild cervids and domestic livestock in farmed landscapes.

The threat of generalist helminth transmission between domestic and wild ruminants is heightened by the growing issue of anthelmintic resistance (AR). Common livestock helminth infections are increasingly difficult to control [1], resulting in production losses, animal welfare issues and potentially increased greenhouse gas emissions. Estimates suggest that helminth infections cost the European livestock industry 1.8 billion Euros annually, with growing costs of AR through ineffective treatments [2]. While AR is of quantifiable economic importance to commercial farms, the livelihoods of resource-poor subsistence farmers can also suffer, as they frequently live in areas with high wild ruminant diversity and are less able to invest in biosecurity measures [3]. The persistence of anthelmintic-resistant helminths in the environment, and in wild or domestic ruminant populations, will depend on dynamic interactions between host, parasite, climate and landscape variables (figure 1). Changes in land cover such as forest fragmentation can result in ruminants such as roe deer living on the periphery of farmland in closer contact with livestock [4,5]. Such changes could alter the diversity of the helminth fauna in both wild and domestic ruminants, including the propagation, maintenance and transfer of drug-resistant genotypes.

Transmission of helminths, including anthelmintic-resistant strains, has been demonstrated from domestic to wild ruminants *in situ* [6], and from wild to domestic ruminants under field and experimental conditions [6,7]. These studies focused on the abomasal nematode *Haemonchus contortus* in deer, but it remains unknown whether wild ruminants commonly experience spill-over infection with livestock helminths, how long any spill-over infections can persist in wild hosts, and how often infection spills back into livestock. Further, it is unknown if wildlife acts as a vector for anthelmintic-resistant helminths between farms, or as an untreated source of *refugia* for anthelmintic susceptible (AS) helminths [8]; or the extent to which parasite life-history influences the likelihood of either outcome. The answers to these questions are likely context-dependent, defined by local variations in climate, host and landscape. Logistical challenges associated with monitoring AR in wild ruminants make it difficult to gather direct evidence on a case-by-case basis [6] or over long periods of time due to resource or logistical constraints, including seasonally restricted hunting seasons. New methods may provide opportunities for more in-depth and longitudinal research to address these questions.

**Figure 2.**
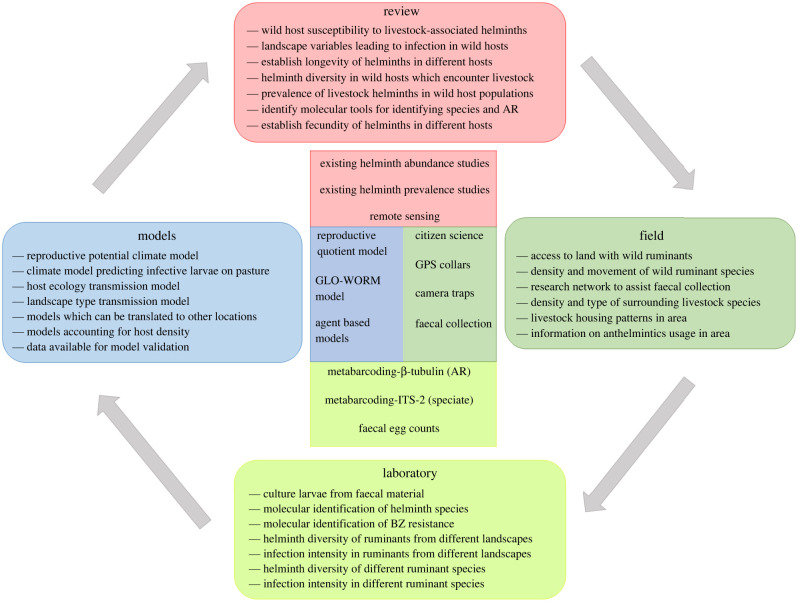
Properties of an ‘ideal’ framework for understanding the role of wild ruminants in AR in livestock, including potential tools for research.

In this review, we explore evidence of cross-transmission of generalist helminths between wild and domestic ruminants, including AR strains, and explore novel methods that will further our understanding of helminth transmission across multi-host landscapes. Relevant literature on laboratory, field and modelling methods are collated, discussed and presented in a framework aimed at shaping future research (figure 2). These aspects of the review are important, as they indicate the most efficient and scalable methods of measuring helminth transmission between wild ruminants and livestock. Our focus is mainly on cervids and other ruminants in Europe, but the principles discussed are likely to apply much more widely across ungulate species assemblages at the wild-domestic interface.

## 2. Generalist helminths and drug resistance in multi-host heterogeneous landscapes

### (a) Cross-transmission and refugia

Wild ruminants share numerous helminths with livestock and the diversity of these in hosts such as European cervids is well researched [9–12]. Despite this, the presence of alleles conferring AR in helminths infecting wild hosts is rarely explored. Generalist helminth species, which are more likely to be transmitted from domestic to wild ruminants [13,14], may contain AR-related alleles; hence in principle drug-resistant nematodes are transferable across the wildlife–livestock interface. Wild ruminants, therefore, could spread-resistant alleles from farms with AR to those without, initiating and accelerating the spread of AR. The consequences of circulation of anthelmintic-resistant genotypes in wild populations are, however, largely unknown, and not necessarily negative. For instance, it is also possible that wild ruminants host *refugia*, which refers to the portion of a helminth population that is not exposed to anthelmintic drugs. This could be particularly evident in situations when *refugia* are rare within farms as a result of treatment practices. Indeed, threats to the viability of anthelmintic drugs are caused by usage that eliminates *refugia* [8]. This includes treating livestock too often and without leaving a proportion of untreated animals large enough to aid the preservation and reintroduction of susceptible alleles [8,15,16]. On farms dominated by a drug-resistant helminth fauna, wild ruminants might act as an important source of *refugia*, providing faeces infected with the eggs of drug-susceptible helminths onto livestock pasture. This could slow the development of AR, as these susceptible parasites from wild hosts interbreed with resistant mutants from livestock and dilute-resistant genotypes. The potential role of wild ruminants as *refugia* for AS livestock helminths is theoretical and has not yet been demonstrated, but is an important consideration when making value judgements of the consequences of cross-species helminth transmission between wild and domestic ruminants.

### (b) Livestock to wild ruminant transmission

The presence of drug-resistant helminths in wild ruminants that are not treated with anthelmintic drugs strongly suggests transmission from livestock, as first recorded in benzimidazole-resistant *H. contortus* in English roe deer [7]. To date, only benzimidazole-resistant nematodes have been identified in wild deer, as mutations conferring resistance to this older class of anthelmintic drug are well-defined and identifiable in nematodes [17,18]. Resistance to other related anthelmintic drugs such as triclabendazole has also been demonstrated with increasing frequency in the generalist liver fluke species *Fasciola hepatica* in livestock [19]. Drug-resistant liver fluke is more difficult to detect in wild ruminants, however, as the genetic loci conferring resistance are not as thoroughly understood compared to benzimidazole resistance in nematodes [20], while the molecular basis of nematode resistance to other drug classes is also less well understood and therefore difficult to measure [21]. Currently therefore, gastro-intestinal nematodes (GINs) offer the best target for identifying the transmission of anthelmintic-resistant helminths from livestock to wild hosts, especially those resistant to benzimidazoles.

Detailed investigation of AR transmission at the landscape level and *in situ* is limited, but has been demonstrated in Hungary. *Haemonchus contortus* extracted from roe deer had 17.1% homozygous resistant alleles, while *H. contortus* extracted from red deer had no drug-resistant alleles [21,22]. Such cross-sectional experiments demonstrate the concept of transmission from domestic to wild ruminants but are limited by seasonal sampling bias. Indeed, roe deer were sampled in spring and summer when *H. contortus* infection is more common [23], whereas red deer were sampled in autumn and winter. Further, substantial longitudinal data are ultimately required to examine the persistence of AR alleles between host populations *in situ*. Collecting adult GINs over long periods is difficult, however, as hunting seasons are typically limited for wild ruminant species [24], and hunters often remove gastro-intestinal material on site [25], making collection and analysis difficult. Advances in metabarcoding are providing opportunities to overcome these obstacles by enabling nematode speciation and AR status identification [26] on faecal material, without the need to hunt wild hosts.

### (c) Wild ruminant to livestock transmission

Demonstrating the origin of anthelmintic-resistant helminths in livestock *in situ* is difficult, as they could result from anthelmintic treatment on site, or alternatively could be introduced by arriving domestic or sympatric wild hosts. Resistant *H. contortus* larvae from roe deer faeces have been fed to successfully infect cattle, and subsequently sheep, in which a 60% frequency of resistant alleles was then identified [7], proving that resistant worms of this species are transmissible from wild to domestic ruminants. Transmission of AR is therefore possible from wild to domestic hosts, but estimating the extent to which it occurs in the wild is difficult. In an area where one farm is dominated by anthelmintic-resistant alleles and the surrounding farms are dominated by AS alleles, wild ruminants might play a significant role in altering the spatial densities of helminth genotypes. They could function as a vector of anthelmintic-resistant alleles to the surrounding susceptible farms, while diluting the proportion of resistant alleles in AR dominant farms. On the other hand, in a situation where all farms have similar levels of resistant alleles within a given helminth species, the role of wild ruminants might be less significant. For instance, the helminth fauna they ingest from one farm might have similar proportions of AR alleles to any helminth eggs they deposit on subsequent farms.

Countries such as Romania and Poland have some of the largest populations of wild ruminants in Europe [27,28] and also have high levels of AR in livestock, particularly to benzimidazole-based products such as albendazole [1]. Longitudinal studies in areas with high AR in livestock, and with adjacent wild ruminants, could provide opportunities to explain the complexities of helminth transmission between host species; however, diversity in the AR status of neighbouring farms might provide the most instructive field sites.

Transmission of AR nematodes between wild and domestic hosts is dependent on a multitude of factors (figure 1), with the order and timing of shared grazing crucial to transmission patterns [29]. This in turn is dependent on climate and weather [13]. It seems likely that the role of wild ruminants in the maintenance and propagation of AR in livestock is very context-dependent, highlighting the importance of studying host and parasite ecology in unison when trying to understand cross-species AR transmission.

### (d) Host susceptibility

Captive wild ruminants infected with anthelmintic-resistant helminths provide valuable insights into the susceptibility of different hosts [6], and their involvement in transmission*.* In European mouflon, the ancestor of domestic sheep [30], anthelmintic-resistant *H. contortus* infection persisted longer and was more intense compared to both fallow and roe deer. *Haemonchus contortus* is traditionally associated with ovine hosts like sheep which could explain this notable susceptibility of the closely related mouflons compared to deer. Indeed, when using faecal egg counts (FECs), a common measure of nematode infection in hosts, European mouflon were producing over 20 000 eggs per gram of faeces 77 days after infection while fallow and roe deer were producing no eggs 58 days after infection [6]. With vastly different levels of infection over time between different wild host species, it is likely that the host species composition of an area could considerably alter the rate of generalist nematode transmission (including anthelmintic-resistant genotypes) between farms.

Other nematodes like *Ashworthius sidemi* also present with vastly different infection levels in different hosts. This multi-host haematophagous abomasal nematode brought to Europe by sika deer [31], can infect European bison with thousands of nematodes [32] and cause reduced red blood cell counts in the host. On the other hand, red and roe deer are typically only infected with a few hundred worms and show little or no pathology [33]. Assuming egg outputs correlate with worm burdens in these wild ruminants, the susceptibility of a host species could influence the rate at which they spread the nematodes across the landscape and to livestock farms. *Ashworthius sidemi* has recently been identified in cattle [34], with concerns that it could be highly pathogenic to the domestic ruminant, which is closely related to bison.

Susceptibility to helminth infection can also differ within the same species due to factors such as age, sex, co-infection and host genetics [35–39]. The sex and age of roe deer have been shown to impact their nematode burden in France, with males having heavier infection burdens in addition to fawns and older adults [35]. Further, older male red deer in Spain had higher levels of infection than females or younger deer [36]. It is possible therefore, that deer populations comprising more males, for example, could drive higher rates of helminth transmission, and that the habitat use of high egg shedding groups disproportionately impacts parasite co-transmission across landscapes. Domestic host infection intensity can also vary within the same species. For instance, both males and younger sheep in the Kashmir valley had heavier GIN infections than female and older sheep [37]. As such, the demographic composition of livestock and wild hosts in an area likely plays an important role in determining helminth transmission patterns, and yet is rarely taken into consideration.

Further, research and breeding programmes often aim to produce more resilient livestock, which has been considered an alternative to anthelmintic drug dependence [38]. Indeed, selective within-flock breeding of Merino sheep in Australia has been shown to increase their resistance to GINs [40], and in the UK, the Southdown sheep breed appears to be less resilient to GIN infections than Shetland and Manx Loaghtan breeds [41]. It is plausible, therefore, that landscapes with more resilient domestic hosts, could in turn reduce generalist and livestock-related helminth infections in wild ruminants such as deer.

Infection from one helminth species can also impact the susceptibility of a host to other helminth species. For instance, *H. contortus* infection in sheep can dampen their immune response, in turn facilitating the establishment of *Trichostrongylus colubriformis*, a common pathogenic intestinal parasite of sheep [39]. On the other hand, helminth infection can also result in hosts having increased susceptibility to other pathogens, with immune suppression caused by nematodes facilitating bovine tuberculosis (bTB) infection in African buffalo [42]. The implications of co-infection for parasite transmission are poorly understood in both wild and domestic ruminants, and further work in this area might provide insights regarding helminth transmission between hosts.

### (e) Host ecology

Changes to habitat, climate and landscape can impact wild ruminant proximity to livestock and their likelihood of carrying certain parasites. Roe deer, for instance, respond to forest fragmentation by using substitute habitat like hedgerows and extend their range until a minimum wood requirement is met [5]. Roe deer in France had higher nematode FECs when in close contact with livestock pasture [43], and it has been suggested that they could ingest livestock-associated helminths from legumes and forbs on pasture [43]. It is therefore quite conceivable that roe deer transmit GINs originating from one farm to other farms within their home range. A study in the European Alps indicated widespread transmission of *H. contortus* between domestic and wild hosts including roe deer [44]. Shared haplotype clusters of *H. contortus* were suggestive of regular cross-transmission at the livestock-wildlife interface [44]. Generalist helminths like *H. contortus* with well-established climatic requirements [45] and tools for molecular investigation [46] can be useful tools to help understand the transmission of nematodes at the wild-domestic interface.

Ruminants with large migrations can also be implicated in cross-species nematode transmission [47]. In Kazakhstan, the saiga antelope is suspected of spreading the abomasal nematode *Marshallagia marshalli* between sheep populations hundreds of kilometres apart during its northern migration [48]. In data-limited situations, the use of social and ecological information has proven useful in indicating the transmission of disease between the saiga antelope and livestock [49], and equivalent information could also prove valuable when understanding helminth transmission. For example, if deer hunters provide samples for nematode analysis, follow-up surveys could ask them where the deer graze and if they are in close proximity to livestock pasture. Comparing nematode infection data with such ecological data from hunters could provide valuable insights into the transmission of generalist and anthelmintic-resistant nematodes. Trematodes can also be transmitted long distances by wild hosts, with species like *Fasciola magna,* a liver fluke originally from North America, being identified in successive adjacent European countries in livestock and deer [50–53], with red deer migration along the Danube considered key to its transmission [54]. Understanding host ecology is therefore crucial in understanding the transmission of generalist and drug-resistant helminths, and in the absence of advanced ecological research equipment such as geo-positioning system (GPS) tags or camera traps, ecological surveys could be useful to provide data relevant to helminth transmission.

### (f) Helminths as epidemiological indicators

While ecological information about wild hosts could provide insights into helminth transmission, the opposite could also be true. Tracking livestock-related helminths and anthelmintic-resistant alleles could offer ecological information about wild ruminant grazing patterns and their contact with livestock pasture. This in turn could present epidemiological data relevant to the transmission of other pathogens which persist in the environment. For instance, *Ostertagia ostertagi*, an abomasal nematode associated with cattle, was present in 70% of sampled roe deer which grazed in an area of intensive cattle farming in England [7], suggesting high levels of contact. Deer have been considered bio-indicators for other pathogens also, with roe deer in Germany being identified as potential indicators of antimicrobial-resistant bacteria in the environment [55]. In fact, there is growing evidence of wildlife becoming infected with drug-resistant bacteria originating from livestock [55–57]. Using a similar concept, infection of deer with livestock-associated helminths could provide valuable insights about their grazing behaviour around livestock and any associated infections to which this could lead. Deer can be infected with multiple bacterial and viral infections that can also infect livestock. For instance, bovine viral diarrhoea virus has been identified in red deer in close proximity to cattle in Spain [58]. In Ireland, sika deer have been infected with bTB from cattle [59,60], while pathogenic and antimicrobial-resistant *Escherichia coli* has been found in red–sika deer hybrids in the same area [61]. Understanding the helminth fauna of wild ruminants, therefore, particularly in areas with other important multi-host pathogens, could present an opportunity to better understand host populations’ wider epidemiological role in the environment.

## 3. Recent research advances and future opportunities

With growing examples of wild ruminants harbouring drug-resistant nematodes from livestock, stimulating more research in this area is important, and increasingly possible with the development of non-invasive advanced molecular and modelling tools. Commonly used methods, such as morphologically identifying adult nematodes or using individual polymerase chain reaction (PCR) assays to recognize anthelmintic-resistant genotypes, lack the efficiency and scalability to monitor year-round changes in helminth fauna in wild ruminant populations and offer limited epidemiological insights into the role of wild ruminants in AR in livestock. Advances in molecular biological techniques have made it possible to identify multiple species [26,62] including anthelmintic-resistant genotypes [63,64] in pooled larval samples after hatching the eggs from faecal material. This provides opportunities for robust longitudinal monitoring and surmounts the issue of requiring adult nematodes only accessible during hunting seasons. In data-limited situations, models can be used to establish likely infection patterns between host species under different landscape, host density and climate scenarios. Advances in climate-based models have opened doors for predicting helminth spill-over from livestock to wild hosts, with models derived for livestock parasites successfully adapted to address this question in mixed-use systems [65]. In other infectious systems, such as chronic wasting disease in white-tailed deer in Missouri, agent-based models (ABMs) have been used to create and investigate epidemiological scenarios in different landscapes [66]. ABMs have yet to be developed for helminth transmission at the wildlife–livestock interface and opportunities could exist in this space.

### (a) Advances in molecular identification

There are a variety of means to assess the presence of different species and anthelmintic-resistant traits using molecular techniques, but these require prior genetic determination of both species and AR loci. For GINs, the internal transcribed spacer 2 (ITS-2) region of the genome is a highly variable, high copy number site that effectively speciates strongylid nematodes of cattle and can resolve genus level identities of other nematodes [67]. Using second-generation sequencing technologies such as Illumina Mi-Seq and Hi-Seq platforms, ITS-2 amplicons generated using universal primers from mixed samples can be used to generate thousands of sequence reads, which can be bio-informatically sorted to match each sequence to a species of interest, which in turn enumerates the relative abundance of each species in a sample [26,68]. This method, dubbed ‘nemabiome’ for GIN research, has been used to identify cultured larvae of nematodes from wild ruminant faecal material in North America and Europe [69,70]. In North America, 84 of 548 wild ruminant samples had livestock-related nematode species [69], while in Europe an apparently isolated roe deer population harboured livestock-related nematodes including the highly pathogenic *H. contortus* [70]. This adds further evidence that wild hosts can act as reservoirs of economically important helminths which are prone to AR. Nemabiome is also referred to as metabarcoding or amplicon sequencing since a PCR product or amplicon is produced prior to sequencing, with the sequences effectively acting as barcodes to identify a species or target of interest [26]. A benefit of amplicon sequencing is the ability to potentially run hundreds of samples together in a single run by sample indexing, which could significantly reduce the cost and increase the throughput of samples.

Measuring anthelmintic-resistant mutations could offer another layer of epidemiological insight and provide evidence that any livestock-related nematodes infecting wild hosts originated from livestock pasture. Metabarcoding can screen for polymorphisms in isotype-1 β-tubulin at codons 167, 198 and 200, which are associated with benzimidazole drug resistance [17,18,46]. Metabarcoding can screen for these mutations in pooled samples [63], whereas until recently the traditional approach required allele-specific PCRs for each polymorphism. To our knowledge, drug-resistant nematodes have not been identified in wild ruminants using metabarcoding, with any identification only occurring through allele-specific PCR in *H. contortus* in roe deer [7,21,22]. The value of using metabarcoding to identify AR nematodes, however, has been demonstrated in sheep in the UK [71] and cattle and bison herds in North America [64]. In the UK, 22 of 174 sheep farms identified mutations at codon 200 of *Nematodirus battus* nematodes using metabarcoding, albeit usually at low individual frequency [71]. In North America, mutations at codon 200 were found with low frequency in cattle-related parasites, highlighting that benzimidazole resistance also has the potential to emerge in bovine hosts [64]. Greater application of advanced molecular tools including the identification of anthelmintic-resistant nematodes in wild ruminants could significantly further our understanding of wild hosts as a vector for anthelmintic-resistant helminths or a source of *refugia* for drug-susceptible helminths.

### (b) Epidemiological modelling

Epidemiological models for helminths generally aim to quantify the levels of infection on pasture and/or in hosts based on a population's relationship with hosts and/or the surrounding environment. These macro-parasite models, such as the GLOWORM-FL framework, are typically designed to forecast infections in livestock but have also been successfully retrofitted to estimate infection spill-over from livestock to wild ruminants. GLOWORM-FL predicts the seasonal availability of free-living infective stages of well-studied nematodes [45] like *H. contortus* on pasture, by including parameters such as the development rate of eggs to infective larvae and larval mortality rates [45]. Related models like the reproductive quotient (Q_0_) model calculate the reproductive potential of nematodes under different environmental conditions by estimating the number of adult female worms produced by one female during its lifetime [72]. The reproductive quotient (Q_0_) model has been used in Botswana alongside GLOWORM-FL to estimate GIN spill-over between zebra, wildebeest and livestock [65] while also showing that strategic timing of anthelmintic treatment could reduce spill-over to wild hosts.

Helminth models are rarely used at the wildlife–livestock interface, despite many existing for wildlife or livestock separately. This is likely because some parasites models are specific to either wild or domestic hosts, such as the thermal suitability model for the reindeer brain-worm *Elaphostrongylus rangiferi* [73]. Other models, however, do include species that infect both wild and domestic ruminants but are rarely used to predict wildlife infection. For instance, *Nematodirus battus* is a highly pathogenic small intestine nematode for sheep which can also infect deer [74,75]. Well-validated air and soil temperature models are frequently used to predict infection in sheep [76], but as the nematode is not obviously pathogenic in wild ruminants, nor are wild ruminants thought to be significant sources of spill-over infections for livestock, there is little stimulus to use the model for wild hosts. With increasing AR, however, and the lack of information about transmission between wild and domestic hosts, expanding climate-driven helminth models for use in wildlife could be important. Other models such as a joint hydro-epidemiological liver fluke model have accurately reproduced *F. hepatica* infection in livestock by integrating hydro-meteorological and parasite models [77]. Despite wild ruminant fluke infections being influenced by surrounding livestock [78], fluke models have not yet been used to investigate such phenomena. Similar to the other examples, the hydro-epidemiological liver fluke model does represent a significant research advancement, but lacks the structure to include stochastic features such as wildlife movement patterns, which will ultimately affect transmission between wild and domestic hosts.

Research from human parasitology could provide suitable concepts for developing models which measure the spread of drug-resistant nematodes between wildlife and livestock. For instance, ABMs have been used to investigate the mechanisms leading to increased helminth infection aggregation in school-aged children after mass anthelmintic drug administration [79]. The model includes age-dependent infection rates across 1000 simulated villages and determined that compliance with drug administration programmes was crucial in eliminating *Necator americanus*, a hookworm that infects humans. An equivalent veterinary model might include species-dependent infection rates with livestock-related helminths, or determine how species movement patterns impact transmission. For example, roe deer are typically isolated territorial mammals, with a small home range [80], whereas fallow deer typically herd by sex, and have a larger home range [81]. Incorporating such detail into existing veterinary helminth transmission models is difficult, but ABMs could provide this opportunity. With drug resistance being an emerging concern in human helminth infections [82,83], any developments in veterinary drug-resistant models could in turn provide a platform for future human parasitology research. Other ABMs have suggested intervention strategies in the transmission of the cestode *Taenia solium* [84], which infects humans and can lead to neurological disease [85]. The model uses a scenario-based approach to suggest that joint drug treatment of humans and vaccination of pigs over 4 years, with 75% coverage, could lead to an increased probability of infection elimination. Scenario-based approaches could be useful when determining drug-resistant helminth infection between wild ruminants and livestock, and provide opportunities for model validation.

### (c) Opportunities for research and understanding using combined methodologies

Using metabarcoding techniques at a local scale over long periods of time could indicate the relative role of wild ruminants in acquiring and transmitting helminths including anthelmintic-resistant genotypes to livestock. Year-round monitoring of wild ruminant nematode fauna, using metabarcoding, could offer important epidemiological insights and provide opportunities to explore the relationship between FEC and nematode species diversity along the gastro-intestinal tract of different wild hosts. This in turn could indicate their susceptibility to livestock-related helminths and their likelihood of transmitting these to other farms. Seasonal trends in FECs have been recorded in wild red deer [86] and in farmed red deer [87]. Understanding how different nematode species impact FEC could further enable our understanding of the extent of infection seasonality and provide opportunities to identify extrinsic and intrinsic factors that influence helminth infection in wild hosts. Attaching GPS tags to wild ruminants has proven useful in assessing the transmission risk of parasites and pathogens that can persist in the environment [88] and for assessing the uptake of GINs relative to the surrounding livestock population [33]. Coupling GPS with advanced molecular techniques in different regions could highlight how landscape factors like habitat fragmentation impact wild host grazing and herding patterns, and subsequently how these patterns impact helminth transmission between livestock farms.

Future epidemiological models could examine the role of wild ruminant grazing and herding behaviours, and help determine how these impact helminth transmission in the context of wider host populations and landscape parameters. ABMs provide opportunities to explore these factors, but could be difficult to validate as helminth fecundity, longevity and establishment rate in different wild hosts is not widely available. Although ABM validation is a common concern across disciplines [89], metabarcoding of larvae for speciation and for identifying drug-resistant alleles, could provide data-rich opportunities for validating scenario-based model outputs. Such validation could be used to infer the susceptibility of different wild ruminant species to livestock-associated helminths, which in turn could be fed back into epidemiological models to provide validation. Furthermore, livestock-related helminth models such as the reproductive quotient (Q_0_) and GLOWORM models could be further utilized, and geographically explicit outputs from such models, could be compared with existing wild ruminant helminth infection data, to determine if relationships exist between climate and livestock-related helminths such as *H. contortus* in wild hosts.

## 4. Conclusion

It is well established that anthelmintic-resistant helminths can spread between wild and domestic hosts [7,21,22] but only domestic to wild transmission has been shown *in situ* [6]. Understanding the susceptibility of different wild hosts and their movement patterns across landscapes could improve our understanding of anthelmintic-resistant nematode transmission between farms [7,33]. Advances in sequencing technology are allowing rapid molecular identification of helminth species [44,45] and their drug-resistant status [63] which is opening up new possibilities for longitudinal research at a local scale and providing increased opportunities for validating complex epidemiological models. Advances in epidemiological modelling are also allowing further investigation of livestock-associated nematodes in wild ruminants and the potential role of livestock contact in the spread of generalist nematodes between wild and domestic hosts [65]. Longitudinal research is required for a deeper understanding of the role of wild ruminants in AR in livestock, and using GPS tags on wild ruminants could indicate patterns and challenge model predictions [33] while providing useful data relevant for other multi-host environmentally persistent pathogens. Modelling can further be extended to explore consequences of climate and land-use change, including altered farm landscapes, for helminth and AR dynamics in the future.

## Data Availability

This article has no additional data.
